# Refined Transgenic Mouse Models Which Recapitulate the Natural Features of Chronic Wasting Disease With Rapid Prion Disease Onsets

**DOI:** 10.1093/infdis/jiaf529

**Published:** 2025-10-16

**Authors:** Joseph P DeFranco, Sehun Kim, Zoe N Atkinson, Jenna Crowell, Samantha Lei, Jifeng Bian, Glenn C Telling

**Affiliations:** Prion Research Center, Department of Microbiology, Immunology, and Pathology, Colorado State University, Fort Collins, USA; Prion Research Center, Department of Microbiology, Immunology, and Pathology, Colorado State University, Fort Collins, USA; Prion Research Center, Department of Microbiology, Immunology, and Pathology, Colorado State University, Fort Collins, USA; Prion Research Center, Department of Microbiology, Immunology, and Pathology, Colorado State University, Fort Collins, USA; Virus and Prion Research Unit, National Animal Disease Center, Agricultural Research Service, United States Department of Agriculture, Ames, Iowa, USA; Virus and Prion Research Unit, National Animal Disease Center, Agricultural Research Service, United States Department of Agriculture, Ames, Iowa, USA; Prion Research Center, Department of Microbiology, Immunology, and Pathology, Colorado State University, Fort Collins, USA

**Keywords:** chronic wasting disease, prions, strains, gene-targeted mice, transgenic mice

## Abstract

Prions are infectious, host encoded proteins which cause fatal neurodegenerative diseases of mammals. Seminal studies showed that overexpression of cervid prion protein (PrP) transgenes eliminated the transmission barrier to chronic wasting disease (CWD) prions in mice. Subsequent models which controlled expression from targeted alleles of the mouse PrP gene provided an improved framework for reproducing additional aspects of natural CWD. Here, we generated mice which combine the advantages of transgene overexpression with refinements afforded by gene targeting. Disease was characterized by accelerated onsets following peripheral or intracerebral challenges and by faithful recapitulation of native CWD strain properties including lymphotropic replication.

## Teaser

We produced mice that over express cervid PrP from microinjected transgene arrays in a genetic background where the endogenous prion protein gene also expresses cervid in place of mouse PrP. These mice had accelerated disease onsets in response to infection with CWD prions, were susceptible to peripheral as well as intracerebral infections, and recapitulated the strain features of naturally occurring CWD prions.

Prion diseases, including human Creutzfeldt–Jakob disease (CJD), bovine spongiform encephalopathy, and scrapie in small ruminants, are a group of invariably fatal and incurable transmissible neurodegenerative disorders [1]. Chronic wasting disease (CWD) is a burgeoning and highly contagious prion disease of cervids in North America, Northern Europe, and South Korea. It is the only known prion disease affecting both wild and captive animals and consequently poses significant threats to public health, agriculture, and wildlife [2]. Prions are composed of PrP^Sc^, a misfolded isoform of the host-encoded cellular form of prion protein, PrP^C^ [1]. Despite lacking informational nucleic acids, prions nonetheless exhibit heritable and mutable strain phenotypes that control disease outcomes. This is accomplished by a unique mechanism in which prion strain information, which is enciphered within distinct PrP^Sc^ conformations, is templated onto PrP^C^ in an iterative process that results in exponential prion accumulation and neurodegeneration in the central nervous system (CNS) [1]. In the case of CWD, prion replication also occurs in peripheral tissues including those of the lymphoreticular system and musculature, and this feature is an important aspect of pathogenesis and contagious transmission in natural host species [2].

The production of transgenic (Tg) mice by embryo pronuclear microinjection of transgenes encoding PrP has been a valuable and widespread means of eliminating the barriers to prion transmission otherwise found in wild-type mice [3]. While this approach has provided tractable experimental models in which to study prions causing naturally occurring diseases of humans and animals, it is nonetheless subject to certain limitations. These include uncontrolled PrP^C^ expression levels from randomly integrated transgene arrays with variable copy numbers and the inability to accurately model allelic heterozygosity and peripheral pathogenesis [3]. We previously produced Tg mice that were susceptible to CWD prion infection [4, 5]. Since North American elk express PrP^C^ with glutamate (E) at residue 226 (E-PrP^C^), while deer, reindeer, and moose encode glutamine (Q) at this position (Q-PrP^C^), we produced TgE and TgQ mice which over express E-PrP^C^ or Q-PrP^C^ in the CNS at levels ∼5-fold higher than wild-type mice [4, 5] (Supplementary Table 1). Subsequently developed gene-targeted (Gt) mice which accurately express E-PrP^C^ or Q-PrP^C^ from targeted alleles of the mouse PrP gene (*Prnp*), referred to as GtE^+/+^ and GtQ^+/+^, were designed to mitigate the aforementioned drawbacks of Tg mice [6]. Importantly, GtE^+/+^ and GtQ^+/+^ mice reproduce the native strain properties of CWD prions and recapitulate critical features of natural CWD including susceptibility to peripheral as well as intracerebral challenges and the ability to replicate infectivity in peripheral as well as CNS tissues [6–8].

Seminal studies in Tg mice also revealed that mouse PrP^C^ (M-PrP^C^) prevented the propagation of CJD prions by human PrP^C^ that was overexpressed from microinjected transgenes [9]. Accordingly, TgE and TgQ mice were engineered on a *Prnp*^−/−^ background in which M-PrP^C^ expression was ablated [4, 5]. We reasoned that E-PrP^C^ or Q-PrP^C^ expressed from microinjected transgenes would likewise be resistant to interference in GtE^+/+^ and GtQ^+/+^ mice since they also fail to express M-PrP^C^. Moreover, since previous studies described an inverse relationship between levels of CNS PrP^C^ expression and prion incubation times [6, 10], we anticipated that mice expressing cervid PrP^C^ both from microinjected transgenes and targeted *Prnp* alleles would exhibit accelerated incubation times following peripheral or intracerebral challenges and that they would recapitulate the strain properties of native CWD prions including lymphotropic replication. Since Tg and Gt mice were both produced on an inbred FVB background, our strategy provided the added benefit of achieving these goals without the concerns of off-target genetic effects.

## METHODS

The offspring of matings between TgE and GtE^+/+^ or TgQ and GtQ^+/+^ mice were bred with additional Gt mice to reconstitute homozygosity of the Gt allele. Mice that were both hemizygous for the transgene array and homozygous for the targeted *Prnp* locus were identified by PCR screening and referred to as TgT mice. TgT mice expressing PrP^C^ with glutamate (E) at residue 226 (E-PrP^C^) are referred to as TgTE. TgT mice expressing glutamine (Q) at residue 226 (Q-PrP^C^) are referred to as TgTQ. Mice were intracerebrally or intraperitoneally challenged with North American CWD prions as previously described [6, 8]. Tissues from diseased mice were collected for neuropathological analyses and various biochemical and immunohistochemical assessments of PrP^C^ and PrP^Sc^ as previously described [6, 8]. Additional methodological details are described in Supplementary Materials.

## RESULTS

**Figure 1.**
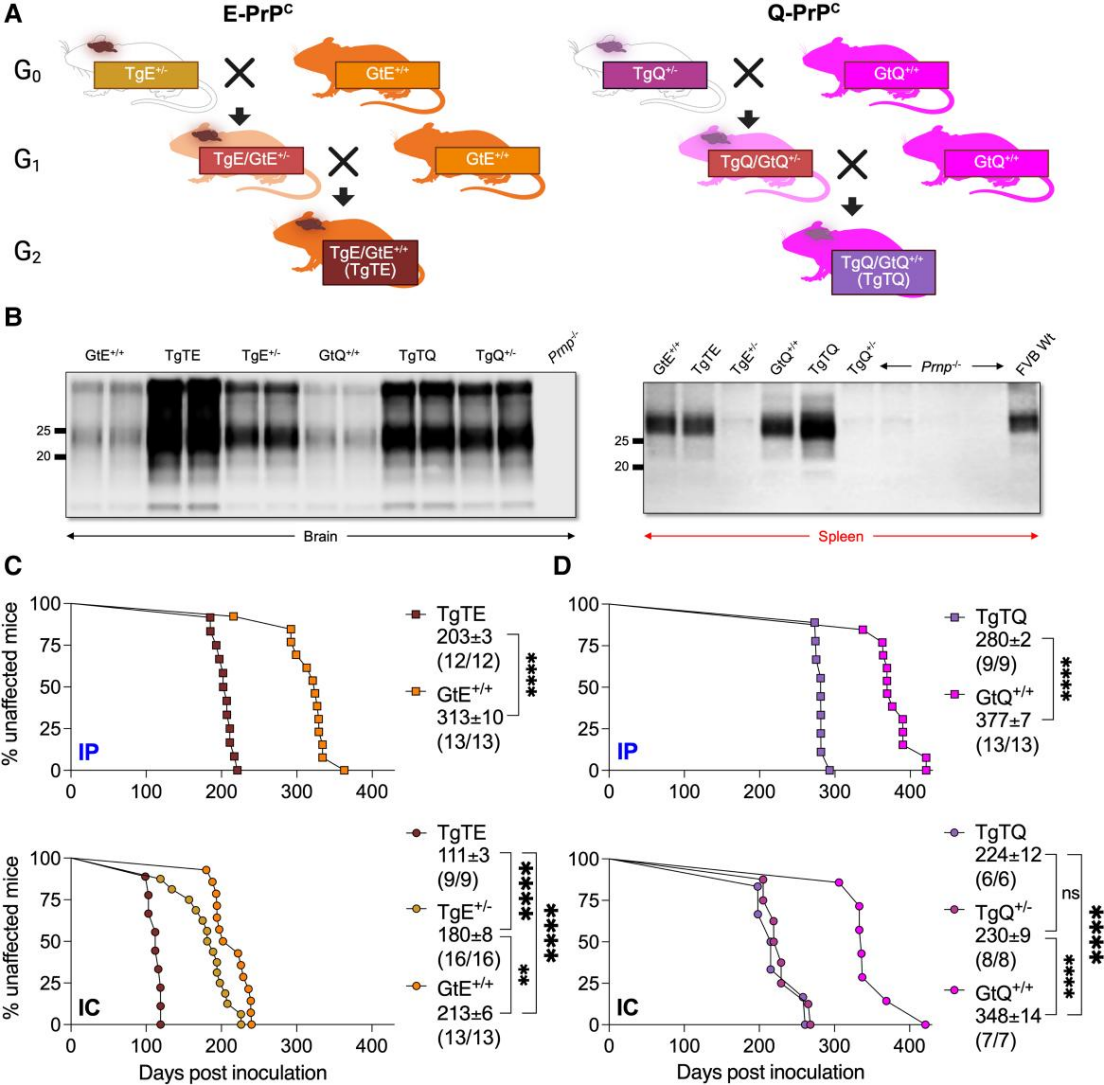
Generation of TgT mice expressing cervid PrP and their responses to CWD prion transmission. *A*, Breeding schemes to produce TgTE mice expressing E-PrP^C^ and TgTQ mice expressing Q-PrP^C^. TgE^+/+^ and TgQ^+/+^ mice homozygous for their respective transgenes were bred with GtE^+/+^ and GtQ^+/+^ mice, respectively. To reconstitute homozygosity of the Gt allele, the resulting mice were bred with additional Gt mice and screened for the transgene using PCR. *B*, Western blotting of brain or spleen homogenates normalized for protein content. The membrane containing brain homogenates was probed with mAb PRC5 and the membrane containing spleen homogenates was probed with mAb Sha31. Molecular weight (kDa) markers are shown. *C* and *D*, Disease kinetics in response to inoculation with North American elk CWD prions. Incubation times are expressed as the mean ± SEM in days with (n/n_0_) indicating the number of diseased mice/number of inoculated mice. Mice were inoculated either intracerebrally (ic, circles) or intraperitoneally (ip, squares). *C*, Mice expressing E-PrP^C^: TgTE (brown), TgE^+/−^ (light brown), GtE^+/+^ (orange). *D*, Mice expressing Q-PrP^C^: TgTQ (purple), TgQ^+/−^ (maroon), GtQ^+/+^ (magenta). Significance between incubation times was determined using the Mantel-Cox test: ****, *P ≤ .0001*; **, *P ≤ .01*; and ns, *P > .05*. Times to disease onset are summarized in Supplementary Table 2, and times to terminal stage of disease are summarized in Supplementary Table 3.

Our previous studies demonstrated that Tg or Gt mice expressing E-PrP^C^ (cervid PrP encoding glutamate at residue 226) had faster disease onsets than their counterparts expressing Q-PrP^C^ (cervid PrP encoding glutamine at residue 226) [6, 11]. We therefore expected that TgTE mice resulting from crosses between TgE and GtE^+/+^ mice would have the fastest CWD prion incubation times (Figure 1*A*). TgTE mice expressed PrP in the CNS at levels ∼6-fold higher than wild-type and GtE^+/+^ mice, while PrP expression in the spleen was equivalent to that found in wild-type and GtE^+/+^ mice (Figure 1*B*; Supplementary Fig. 1 and Table 1). Intraperitoneal challenges of TgTE mice with CWD prions produced disease after 203 ± 3 days (±SEM), which represented a 35% reduction compared to the 313 ± 10 days mean incubation time in GtE^+/+^ mice (Figure 1*C*; Supplementary Tables 2 and 3). Intracerebrally inoculated TgTE mice developed disease after 111 ± 3 days, which was faster than the previously reported 124 ± 6 days incubation time in intracerebrally inoculated TgE mice that were homozygous for the transgene array [6]. Intracerebral challenges produced disease in hemizygous TgE mice after 180 ± 8 days and after 213 ± 6 days in GtE^+/+^ mice (Figure 1*C*; Supplementary Tables 2 and 3). We conclude that co-expression of E-PrP^C^ from both the transgene array and from targeted *Prnp* alleles produced accelerated incubation times compared to progenitor TgE and GtE^+/+^ mice.

TgTQ mice resulting from crosses between TgQ and GtQ^+/+^ mice (Figure 1*A*) expressed PrP in the CNS at levels ∼6-fold higher than wild-type and GtQ^+/+^ mice, while PrP expression in the spleen was equivalent to that of wild-type and GtQ^+/+^ mice (Figure 1*B*; Supplementary Fig. 1 and Table 1). Intraperitoneal inoculation of TgTQ mice produced disease after 280 ± 2 days (Figure 1*D*; Supplementary Tables 2 and 3). While this incubation time was ∼80 days longer than intraperitoneally inoculated TgTE mice, it was ∼100 days faster than intraperitoneally challenged GtQ^+/+^ mice and ∼230 days more rapid than intraperitoneally challenged TgQ mice (Figure 1*D*; Supplementary Tables 2 and 3). Intracerebral inoculation of TgTQ mice produced disease after 224 ± 12 days, while disease in hemizygous TgQ and GtQ^+/+^ mice occurred after 230 ± 9 days and 348 ± 14 days respectively (Figure 1*D*; Supplementary Tables 2 and 3). The accelerated responses of TgTE compared to TgTQ mice support our previous findings that amino acid variation at residue 226 of cervid PrP^C^ influences the kinetics of CWD prion replication (Supplementary Table 2) [6, 8].

**Figure 2.**
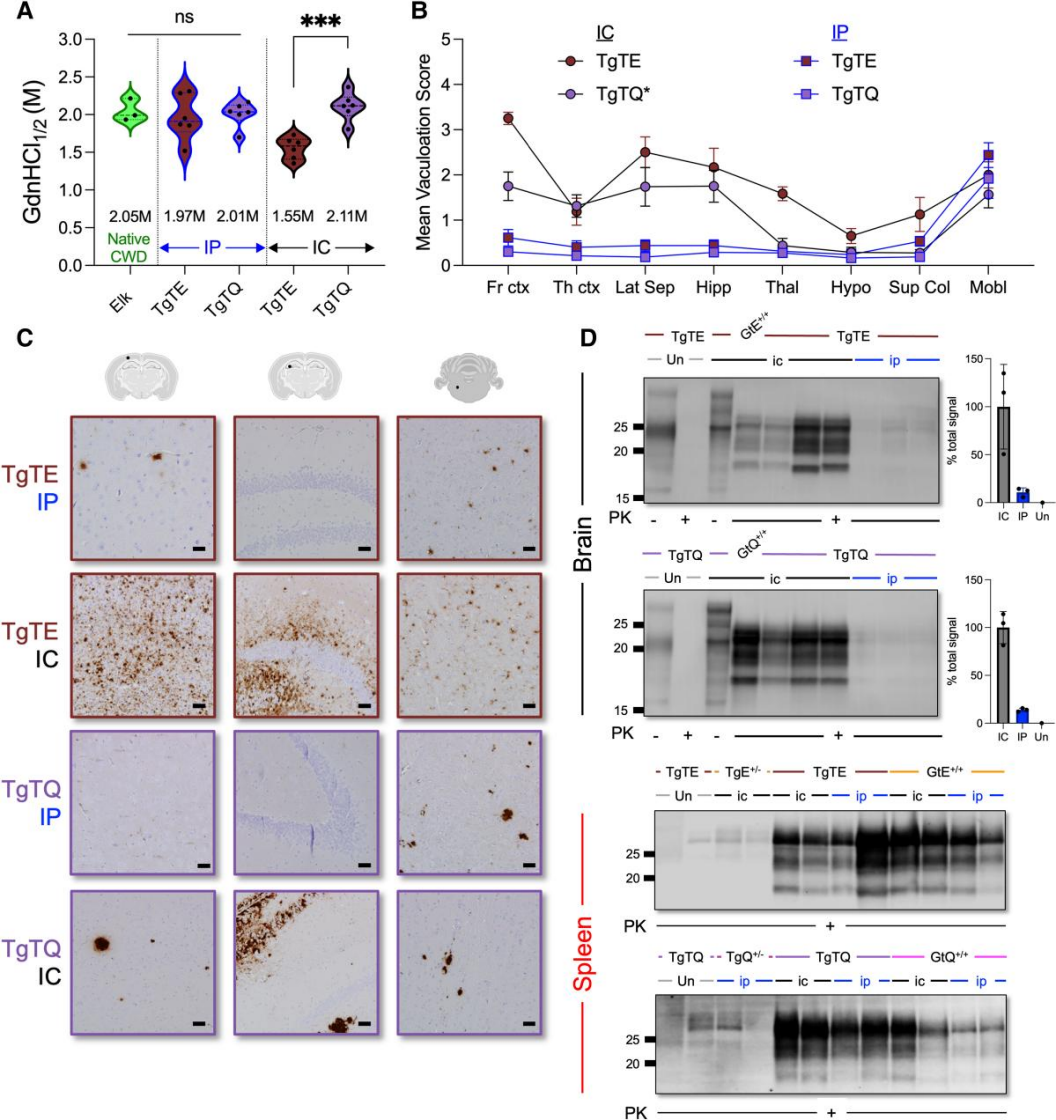
Disease outcomes in CWD-inoculated TgTE and TgTQ mice. *A—D*, Analyses of diseased TgTE (brown) and TgTQ (purple) mice following intracerebral (ic) or intraperitoneal (ip) transmissions of NA CWD prions. *A*, Conformational stabilities of NA elk CWD prions (99W12389) and their derivatives following intracerebral or intraperitoneal challenges of TgT mice shown as [GdnHCl_1/2_] values. For each condition, individual replicates are shown as points in the violin plots from three technical replicates of native elk CWD brain tissue and three biological replicates from two independent experiments in TgT mice. *B*, Assessments of spongiform degeneration in the CNS of diseased TgT mice. Fr ctx, frontal cerebral cortex; Th ctx, cerebral cortex at the level of the thalamus; Lat Sep, lateral septal nucleus; Hipp, hippocampus; Thal, thalamus; Hypo, hypothalamus; Sup Col, superior colliculus; Mobl, medulla oblongata. Data points represent mean scores ± SEM for four mice per group. Lesions in the brains of intracerebrally inoculated TgTQ(*) mice were asymmetrically distributed and the scores from both hemispheres were averaged before plotting. *C*, Immunohistochemical analyses of disease-associated prion protein in forebrain or hindbrain brain regions of diseased TgT mice. Images taken in the somatosensory cortex, Ammon's horn of the hippocampal region, and motor-related region of the medulla. Scale bar = 50 μM. *D*, Immunoblotting of brain or spleen homogenates from uninfected (Un) or diseased TgT, Gt, or Tg mice. Molecular weight (kDa) markers are shown. Homogenates were either treated (+) or not treated (−) with PK as indicated. For immunoblotting of brain homogenates, average fractions of total signals are plotted ± SD and individual replicates are shown as dots. In *D*, signals from intracerebrally inoculated TgT mice were averaged and arbitrarily set as 100%. Signals from intraperitoneally inoculated TgT mice were averaged and expressed as a fraction of the intracerebrally inoculated signal. Background was assessed in uninfected TgT homogenate lanes.

We monitored the conformational properties of CNS CWD prions in diseased TgTE and TgTQ mice by progressive denaturation of PrP^Sc^ with guanidine hydrochloride (GdnHCl) [6]. The denaturation profiles and GdnHCl concentrations producing half-maximal denaturation of PrP^Sc^ ([GdnHCl_1/2_]) in the brains of intraperitoneally challenged TgTE and TgTQ mice were indistinguishable from PrP^Sc^ comprising CWD prions in elk brain homogenate (Figure 2*A*; Supplementary Fig. 2A). By contrast, intracerebral inoculations produced divergent PrP^Sc^ conformational profiles and [GdnHCl_1/2_] values in the brains of TgTE and TgTQ mice (Figure 2*A*; Supplementary Fig. 2B). These findings extend our previous findings showing that residue 226 influences the conformational properties of CWD prions in intracerebrally challenged Tg and Gt mice and that different routes of inoculation result in the selective propagation of distinct CWD prion strain conformers in Gt mice [8, 11].

In light of these results, we explored whether different routes of inoculation produced additional CWD prion strain differences in TgT mice. Intraperitoneal and intracerebral challenges with CWD prions produced distinct clinical signs (Supplementary Table 4). Neuropathological assessments revealed equivalent severities and distributions of neuronal vacuolation in intraperitoneally inoculated TgTE and TgTQ mice, with hindbrain regions exhibiting the most pronounced spongiform degeneration (Figure 2*B*). Immunohistochemical analysis showed that PrP aggregates were equally prominent in the medulla of intraperitoneally inoculated TgTE and TgTQ mice and to a lesser extent in the cortex and hippocampus (Figure 2*C*). These presentations broadly resemble our previous findings showing indistinguishable neuropathological profiles in intraperitoneally inoculated GtE^+/+^ and GtQ^+/+^ mice [8]. By contrast, neuropathology in intracerebrally inoculated TgT mice was more severe and was influenced by differences at residue 226 (Figure 2*B*). While forebrain regions of TgTQ mice contained asymmetrically distributed florid plaques (Supplementary Fig. 3), neuronal vacuolation was symmetrically distributed and more severe in the frontal cortex and thalamus of intracerebrally inoculated TgTE mice. Disease-associated PrP accumulated in dense, coarse aggregates in the cortex and hippocampus of intracerebrally inoculated TgTQ mice, while deposition patterns in TgTE mice were a mixture of punctate aggregates and diffuse staining (Figure 2*C*). Western blotting of proteinase K (PK) treated brain homogenates revealed elevated PrP^Sc^ levels in intracerebrally inoculated TgTE and TgTQ mice compared to intraperitoneally inoculated counterparts (*P* ≤ .05 and *P* ≤ .001, respectively; Figure 2*D*). Collectively, these findings show that peripheral and intracerebral challenges of TgT mice result in the selective propagation of distinct CWD prion strains which produced distinct disease outcomes. These observations are concordant with our previous findings in Gt mice [8].

We previously showed that while the spleens of GtQ^+/+^ mice accumulated prions during CWD infection, this was not the case in diseased TgQ mice [6]. While western blotting revealed that spleen homogenates of intracerebrally and intraperitoneally inoculated TgT mice contained PK-resistant PrP^Sc^ at levels similar to those detected in Gt mice, we were unable to detect prions in the spleens of diseased Tg mice by western blotting (Figure 2*D*). Immunohistochemical analyses of spleens from intraperitoneally inoculated TgTQ and GtQ^+/+^ mice revealed comparable deposition patterns of disease-associated PrP (Supplementary Fig. 4). These results are reminiscent of previous findings demonstrating PrP^Sc^ accumulation in lymphoid tissues of CWD-infected cervids [12]. We conclude that expression of E-PrP^C^ or Q-PrP^C^ from targeted *Prnp* alleles in peripheral tissues (Figure 1*B*) facilitates splenic replication of CWD prions in TgT and Gt mice.

## DISCUSSION

The development of models that recapitulate the native properties of human and animal prions is essential for monitoring disease transmission of prion strains including their host range potential. Previously generated hybrids of knock-in (Ki) and Tg models, referred to as Ki + Tg mice, used concentrated inocula and employed Ki mice that were variably susceptible to human prions [13]. While Ki + Tg mice expressed human PrP^C^ only from a single targeted *Prnp* locus [13], TgTE and TgTQ mice reported here express cervid PrP from targeted alleles in the homozygous state in the presence of the corresponding overexpressing transgene. This approach resulted in rapid disease models that recapitulate critical aspects of CWD pathogenesis, including prion replication in lymphoreticular tissues. The ∼110 days incubation period in intracerebrally inoculated TgTE mice reported here is, to date, the fastest recorded primary transmission of CWD prions. While previous reports support the existence of multiple CWD strains [7, 11, 14, 15], the strain properties of prions associated with contagious CWD in North America appear to be broadly invariant [6, 8]. It therefore seems likely that intracerebral challenges of TgTE mice could be used as a general means to rapidly assess the transmission properties of North American CWD prions from a variety of sources.

Our findings that different routes of inoculation result in the selection of distinct CWD prion strains in TgT mice reinforce our previous discoveries in Gt mice and implicate the involvement of tissue-specific factors in this process [8]. While peripherally challenged TgT and Gt mice both retain the strain properties of CWD prions observed in native host species, the ∼200 days and ∼280 days incubation periods of intraperitoneally inoculated TgTE and TgTQ mice, respectively, were ∼30% shorter than their Gt progenitors. These findings suggest that TgTE and TgTQ mice will be a useful and rapid model in which to study the transmissibility, host-range potential, and evolution of native prion strains. Finally, our findings support the general feasibility of the TgT strategy for rapid and accurate strain assessments of additional animal as well as human prion diseases.

## Supplementary Material

jiaf529_Supplementary_Data

## Supplementary Data

Supplementary materials are available at *The Journal of Infectious Diseases* online (http://jid.oxfordjournals.org/). Supplementary materials consist of data provided by the author that are published to benefit the reader. The posted materials are not copyedited. The contents of all supplementary data are the sole responsibility of the authors. Questions or messages regarding errors should be addressed to the author.
