# Supplementary material for: Refined Transgenic Mouse Models Which Recapitulate the Natural Features of Chronic Wasting Disease With Rapid Prion Disease Onsets
Source: J Infect Dis. 2025 Oct 16;233(1):e271–6. doi: 10.1093/infdis/jiaf529 (PMC12811869; doi:10.1093/infdis/jiaf529)
Supplement: jiaf529_Supplementary_Data [file jiaf529_supplementary_data.docx]

**Supplementary Materials for:**

**Refined transgenic mouse models which recapitulate the natural features of chronic wasting disease with rapid prion disease onsets**

Joseph P. DeFranco, Sehun Kim, Zoe N. Atkinson, Jenna Crowell, Samantha Lei, Jifeng Bian, and Glenn C. Telling^*^

* Corresponding author to whom correspondence should be addressed:

Email: [glenn.telling@colostate.edu](mailto:glenn.telling@colostate.edu)

**Supplementary Materials and Methods**

**Ethics Statement** Animal work was performed in an AAALAC accredited facility in compliance with the Guide for the Care and Use of Laboratory Animals. All procedures used in this study were performed in compliance with the Colorado State University Institutional Animal Care and Use Committee.

**Animal Models** Tg5037 mice, referred to here as TgE mice, were previously generated by microinjection of the MoPrP.Xho expression construct containing the PrP^C^-E coding sequence [5]. Tg1536 mice, referred to here as TgQ mice, were previously generated by microinjection of the cosSHa.Tet expression construct containing the PrP^C^-Q coding sequence [4]. The generation of GtE^+/+^ and GtQ^+/+^ mice has been previously described [6].

TgTE mice were generated by breeding TgE with GtE^+/+^ mice, while TgTQ mice resulted from crosses between TgQ and GtQ^+/+^ mice. TgE and TgQ parents were homozygous for their respective transgene arrays, referred to as TgE^+/+^ and TgQ^+/+^. The resulting F1 hybrids were backcrossed with additional GtE^+/+^ and GtQ^+/+^ mice. TgT mice that were both hemizygous for the transgene array and homozygous for the respective targeted *Prnp* alleles were identified by PCR screening. Screening of offspring was conducted using PCR with the following forward and reverse primer pairs: for the cosSHa.Tet transgene: 5’-CAATGACGTGTTGCTGGAGTAC-3’ and 5’-AGAGCTACGGTGGATAACC-3’ primers; for the MoPrP.Xho transgene: 5’-GAACTGAACCATTTCAACCGAG-3’ and 5’-AGAGCTACAGGTGGATAACC-3’ primers; for GtE^+/+^ and GtQ^+/+^ targeted alleles; 5’-GGCCAGTGGATCAGTATAATAACC-3’ and 5’-TCAATTGAAAGAGCTACAGGTGG-3’ primers.

**Prion transmissions** The frozen brain of a diseased elk referred to as 99W12389 from the United States [11] was homogenized in phosphate-buffered saline (PBS) lacking calcium and magnesium ions (10 % w/v). TgT, Tg, and Gt mice were challenged by intracerebral or intraperitoneal routes of inoculation as previously described [8]. Inoculated Tg mice were hemizygous for their respective transgene arrays unless otherwise indicated. Six- to eight-week-old mice were anesthetized with isoflurane prior to inoculation. For intracerebral inoculations, 30 µl of a 1 % brain homogenate was inoculated into the right parietal cortex. For intraperitoneal inoculations, mice were inoculated with 100 µl of a 1 % brain homogenate. Animals were maintained on an alternating 12-h light and 12-h dark cycle with free access to food and water. Inoculated mice were assessed twice weekly for the appearance of neurological signs consistent with prion disease including truncal ataxia, plastic tail, loss of extensor reflex, loss of pinched tail response, aggressive behavior, hunched posture, head bobbing, head tilting, difficulty in righting from a supine position, slowed movements, circling, balance issues, limb paralysis/disfunction, dull or rough coat, loss of weight, flattened gait, and depressed behavior. The time to disease onset, or incubation time, is the time between the date of inoculation and the first date on which definitive, subsequently progressive clinical signs were identified. Mice were humanely killed at the terminal stage of disease. Incubation time data are summarized in Table S2, while the time to terminal stage of disease for each transmission is summarized in Table S3. Mice dying from intercurrent illnesses were excluded from incubation time calculations. The brains of a subset of diseased mice were removed and bisected down the midline. One hemisphere was frozen for biochemical analyses; the other was immersion fixed in formalin for neuropathological assessments. The whole brains of additional diseased mice were removed and immersion fixed in formalin for additional neuropathological assessments.

**Conformational stability analysis (CSA)** was conducted as previously described [8]. Protein amounts in 10 % (w/v) brain homogenates were assessed using the bicinchoninic acid (BCA) assay (ThermoFisher Scientific™). To normalize PrP^Sc^ signals, brains with higher levels, determined by western blotting, were diluted in brain homogenates from *Prnp^-/-^* knockout mice which do not express M-PrP. Brain homogenates were treated with increasing concentrations of GdnHCl in 96-well plates. After a 1 h incubation at room temperature, samples were adjusted to 0.4 M GdnHCl with PBS. Treated brain homogenates were transferred onto nitrocellulose membranes (Whatman GmbH, Dassel, Germany) using a dot blot apparatus. After two PBS washes, membranes were air dried overnight. After the membranes were rehydrated with PBS, they were incubated with 5 µg/ml PK in cell lysis buffer (50 mM Tris-HCl, pH 8.0, 150 mM NaCl, 0.5 % sodium deoxycholate, and 0.5 % Igepal CA-630) for 1 h at 37 °C. PK was inactivated with 2 mM phenylmethylsulfonyl fluoride (PMSF). Membranes were treated with 3 M guanidine thiocyanate/20 mM Tris-HCl, pH 7.8, for 10 min at room temperature. After four washes with PBS, membranes were blocked with 5 % Bio-Rad™ Blotting-Grade Blocker in Tris-buffer saline containing Tween (TBST) for 1 h. Membranes were probed with mAb PRC5 which recognizes an epitope that includes amino acid residues 135 and 162 in the structured globular domain of PrP [16]. Primary antibody was diluted in TBST 1:5,000 overnight at 4 °C, followed by HRP-conjugated goat anti-mouse IgG secondary antibody (1:5,000) (Amersham™, Cytiva). The membrane was developed with Pierce™ ECL Western Blotting Substrate (Thermo Scientific™) and scanned with an ImageQuant LAS 4000 (GE Healthcare). Signal were analyzed with ImageQuant TL 7.0 software (GE Healthcare).

**Immunohistochemical analyses** were conducted as previously described [8]. Briefly, brains embedded in paraffin blocks were coronally sectioned at 5 µm. Brain sections were placed on positively charged glass slides and heated to 60 °C for 30 min. Slides were treated with xylene and graduated ethanol treatment followed by 88 % formic acid for 30 min. Antigen retrieval was conducted using citrate buffer in the 2,100 Retriever (Proteo-Genix, Schiltigheim, France). Slides were treated with 5 % nonfat milk for 30 min at room temperature followed by Fab D18 diluted at 1:2,500 in PBST overnight at 4 °C. Slides were exposed to biotin-labeled goat Fab anti-human IgG secondary antibody (Southern Biotech, Birmingham, AL) at a 1:2,500 dilution for 1 h at room temperature. Slides were developed using avidin-conjugated horseradish peroxidase (HRP) with diaminobenzidine (DAB) as substrate (Vector Laboratories, Burlingame, CA) for 30 min.

**Lesion profiling**  was performed as previously described [8]. Coronal sections of paraffin-embedded mouse brains were stained with hematoxylin and eosin. Microscopic images of selected regions of the CNS were independently evaluated by two investigators for the severity of vacuolar degeneration in cerebral gray matter. The severity of vacuolation was scored on a scale of zero to five.

**Immunoblotting of PrP^Sc^** Homogenates were normalized for total protein content after BCA analyses. Brain homogenates were treated with 50 µg/ml PK in the presence of 2 % sarkosyl for 1 h at 37 °C. Digestion was terminated with 2 mM PMSF for 10 min at room temperature and samples were denatured in SDS-PAGE loading buffer at 100 °C for 10 min. Spleen homogenates containing 500 µg of total protein were treated with 1 mg/ml DNase and 5mM MgCl_2_ prior to PK digestion with 50 µg/ml PK in the presence of 2 % sarkosyl for 1 h at 37 °C. Digestion was terminated with 2 mM PMSF for 10 min at room temperature and subjected to centrifugation at 100,000 x g for 1 h at 4 °C. The supernatant was discarded and the resulting pellet was resuspended in SDS-PAGE loading buffer and heated at 100 °C for 10 min. Denatured brain and spleen samples were subjected to SDS-PAGE using precast 12 % discontinuous Bis-Tris gels (Bio-Rad Laboratories, Inc.). Proteins were transferred to Immobilon-FL PVDF membranes (EMD Millipore) and blocked with 5 % non-fat milk for 1-2 h. Blots were treated with mAb PRC5 diluted 1:5,000, followed by horseradish peroxidase–conjugated anti-mouse IgG secondary antibody. Membranes were developed by using Pierce™ ECL Western Blotting Substrate (Thermo Scientific™) and band densities were assessed using Fujifilm imaging software.

**Additional Reference for Supplementary Materials and Methods**

16. Kang HE, Weng CC, Saijo E, et al. Characterization of conformation-dependent prion protein epitopes. The Journal of Biological Chemistry **2012**; 287:37219-32.


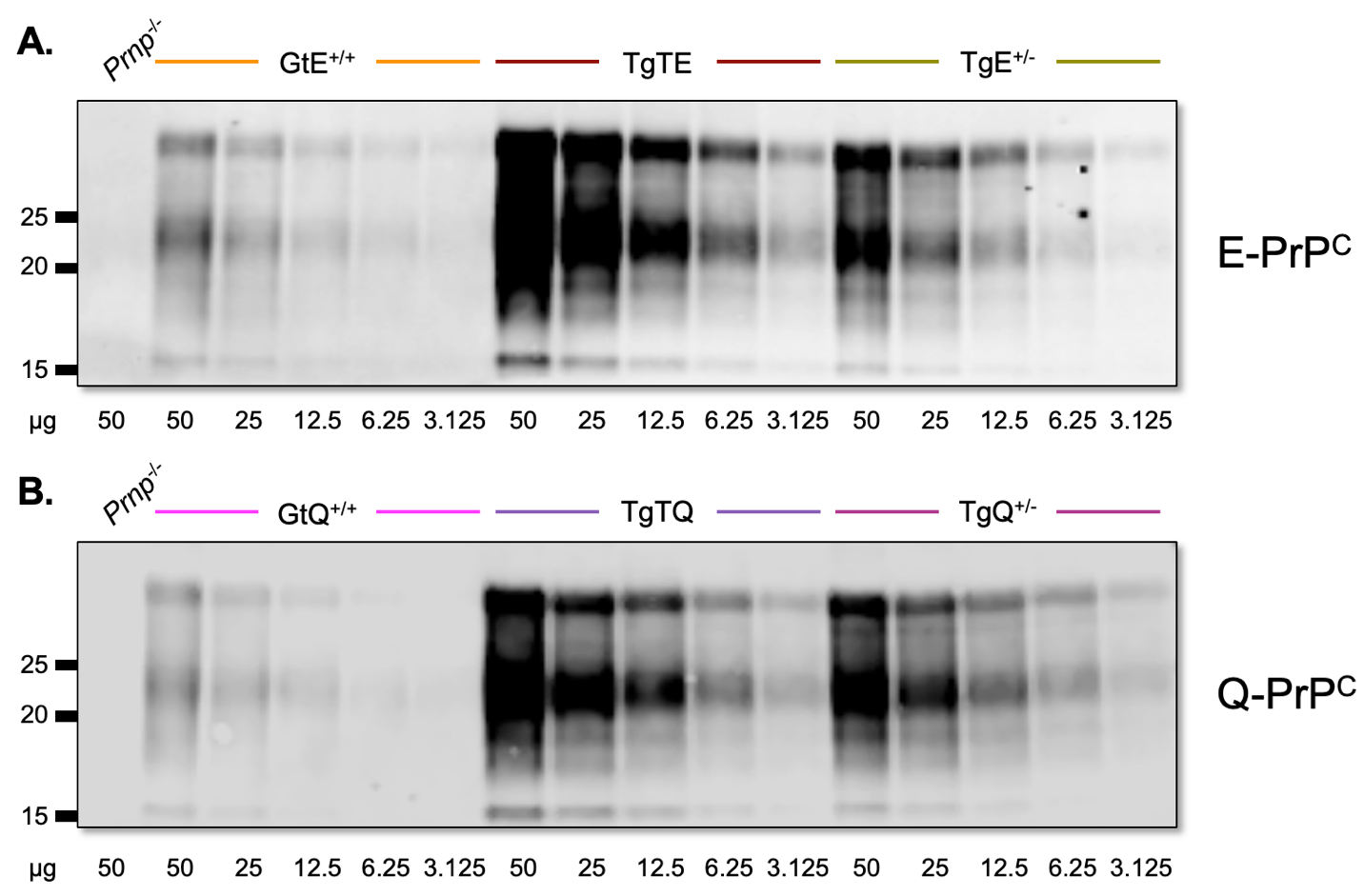


**Supplementary Figure 1: Levels of PrP^C^ expression in the central nervous system of mice engineered to encode cervid PrP.** Levels of PrP^C^ expression in the central nervous system were estimated by western blotting using mAb PRC5. Representative western blots for mice expressing cervid PrP with glutamine (E), termed E-PrP^C^ in **A.**, and mice expressing cervid PrP with glutamine (Q), termed Q-PrP^C^ in **B.** Amounts of total protein loaded (μg) in each sample for different mouse lines are as indicated. The positions of 25, 20, and 15 kDa molecular weight markers are shown.


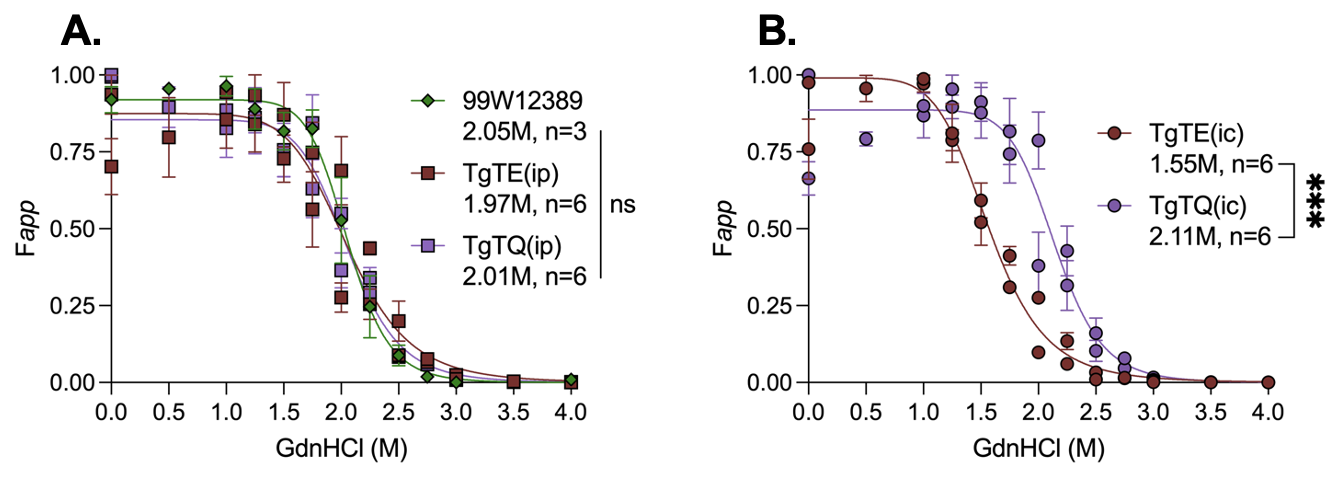


**Supplementary Figure 2: Responses of CWD-infected brain homogenates to increasing concentrations of guanidine hydrochloride (GdnHCl)** Average fractional apparent signal (F*app*) ± SEM after PK digestion is plotted as a function of GdnHCl concentration (M). Sigmoidal dose–response curves were plotted using a four-parameter algorithm. Significance was determined by comparing concentrations of GdnHCl to produce half-maximal denaturation (****, *P ≤* 0.0001; ns, *P >* 0.05). **A.**, elk CWD isolate, 99W12389, green diamonds; intraperitoneally-inoculated TgTE mice, brown squares; intraperitoneally-inoculated TgTQ mice purple squares. **B.**, intracerebrally-inoculated TgTE mice, brown circles; intracerebrally-inoculated TgTQ mice, purple circles. Three technical replicates were conducted for the native elk CWD isolate, 99W12389, and three biological replicates were independently analyzed twice for TgT mice.

**
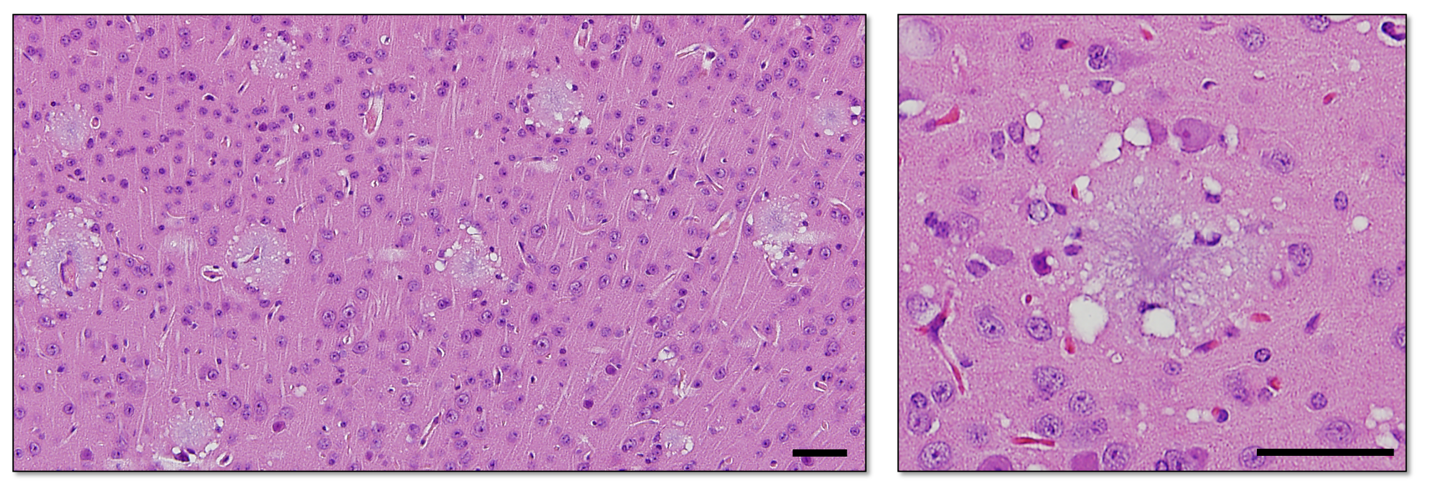
**

**Supplementary Figure 3: Florid plaques in the cerebral cortex of intracerebrally-inoculated TgTQ mice** Microscopic images of hematoxylin and eosin-stained brain sections taken in the hemisphere ipsilateral to the site of inoculation. Scale bar = 50μM.


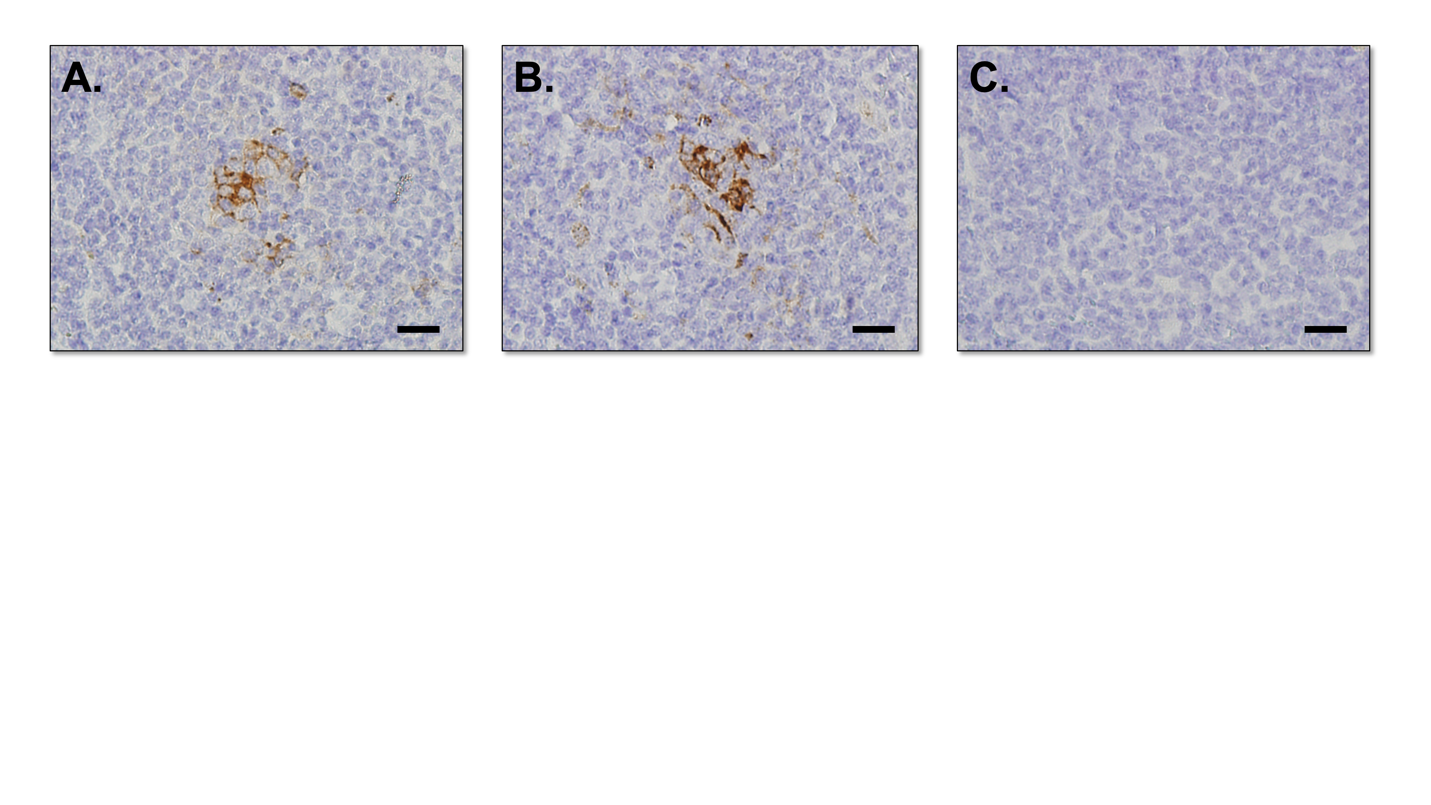


**Supplementary Figure 4: Immunohistochemical analysis of formalin-fixed spleen tissue** Microscopic images of the lymphoid follicles from **A.**, spleen of a diseased TgTQ mouse following intraperitoneal inoculation; **B.**, spleen of a diseased GtQ mouse following intraperitoneal inoculation; **C.** , spleen of an uninfected GtQ mouse. Scale bars = 10μM.

**Supplemental Table 1. Levels of PrP^C^ expression in the brain.**

| GtE^+/+^ | TgTE | TgE^+/-^ | GtQ^+/+^ | TgTQ | TgQ^+/-^ |
| --- | --- | --- | --- | --- | --- |
| Wild type (1X) | ~ 5-7 X | ~ 4-6 X | Wild type (1X) | ~ 5-7 X | ~ 4-6 X |

Levels of total PrP^C^ expression were assessed by western blotting of three mouse brain homogenates from each line. Levels of protein were assessed using BCA. Immunoblots of doubling dilutions of brain homogenates containing known amounts of protein were probed with mAb PRC5. A representative immunoblot is shown in Figure S1. PrP^C^ signals were scanned and averaged between samples, and fold differences compared to Gt mice which express wild type levels of PrP^C^ (designated by X) were calculated.

**Supplemental Table 2. Times to disease of mice inoculated with North American elk CWD (99W12389) prions.**

|  | TgTE | TgTQ | Δ TgTE – TgTQ |
| --- | --- | --- | --- |
| Intraperitoneal | 203 ± 3 (12/12) | 280 ± 2 (9/9) | 28 % (*P* < 0.001) |
| Intracerebral | 111 ± 3 (9/9) | 224 ± 12 (6/6) | 50 % (*P* < 0.0001) |
|  | GtE^+/+^ | GtQ^+/+^ | Δ GtE – GtQ |
| Intraperitoneal | 313 ± 10 (13/13)* | 377 ± 7 (13/13)* | 17 % (*P* < 0.0001) |
| Intracerebral | 213 ± 6 (14/14)* | 348 ± 14 (7/7)* | 39 % (*P* < 0.0001) |
|  | TgE^+/-^ | TgQ^+/-^ | Δ TgE^+/-^ – TgQ^+/-^ |
| Intraperitoneal | ND | 508 ± 12 (7/7)* | ND |
| Intracerebral | 180 ± 8 (16/16) | 230 ± 9 (8/8)* | 22 % (*P* < 0.0001) |

Time to disease onset (incubation time) is expressed as the mean, in days, at which inoculated mice first developed ultimately progressive signs of neurological disease. Variance is expressed as ± standard error of the mean (SEM). n/n_0_, number of diseased mice/number of inoculated mice. Mice dying of intercurrent illnesses prior to prion disease onset were excluded from these calculations. ND, not determined. *, transmissions previously reported in [6, 8].

**Supplemental Table 3. Times to terminal stage of disease of mice inoculated with North American elk CWD (99W12389) prions.**

|  | TgTE | Duration of  clinical phase | TgTQ | Duration of  clinical phase |
| --- | --- | --- | --- | --- |
| Intraperitoneal | 213 ± 3 (12/12) | 10 ± 1 | 287 ± 4 (9/9) | 7 ± 3 |
| Intracerebral | 120 ± 2 (9/9) | 9 ± 3 | 239 ± 18 (6/6) | 15 ± 7 |
|  | GtE^+/+^ | Duration of  clinical phase | GtQ^+/+^ | Duration of  clinical phase |
| Intraperitoneal | 326 ± 10 (13/13) | 16 ± 3 | 393 ± 7 (13/13) | 14 ± 3 |
| Intracerebral | 226 ± 6 (14/14) | 13 ± 3 | 372 ± 15 (7/7) | 18 ± 5 |
|  | TgE^+/-^ | Duration of  clinical phase | TgQ^+/-^ | Duration of  clinical phase |
| Intraperitoneal | ND | ND | 557 ± 20 (7/7) | 50 ± 10 |
| Intracerebral | 192 ± 9 (16/16) | 12 ± 1 | 241 ± 8 (8/8) | 10 ± 1 |

Time to end of clinical phase is expressed as the mean, in days, at which inoculated mice were sacrificed at the terminal stage of neurological disease. Variance is expressed as ± standard error of the mean (SEM). n/n_0_, number of diseased mice/number of inoculated mice. Mice dying of intercurrent illnesses prior to prion disease onset were excluded from these calculations. Duration of clinical phase is the time, in days, between the beginning of clinical onset and the terminal stage of disease. Variance is expressed as ± standard error of the mean (SEM).

**Supplemental Table 4. Comparison of clinical signs of intraperitoneally- or intracerebrally-inoculated TgTE and TgTQ mice at terminal stage of disease.**

| **Specific clinical signs for intracerebrally-inoculated mice** | **Shared clinical signs** | **Specific clinical signs for intraperitoneally-inoculated mice** |
| --- | --- | --- |
| Dramatic head tilt | Truncal ataxia | Abnormal hindlimb control/movements |
| Head bobbing | Plastic tail | Modified gait |
| Circling | Flattened gait | Partial or complete inability for hindlimbs to support body weight |
| Impaired balanced and total body control | Difficulty righting |  |
|  | Loss of extensor reflex |  |
